# An alternate route of ethylene receptor signaling

**DOI:** 10.3389/fpls.2014.00648

**Published:** 2014-11-20

**Authors:** Jingyi Zhang, Jing Yu, Chi-Kuang Wen

**Affiliations:** National Key Laboratory of Plant Molecular Genetics and National Center for Plant Gene Research (Shanghai), Institute of Plant Physiology and Ecology, Shanghai Institutes for Biological Sciences, Chinese Academy of SciencesShanghai, China

**Keywords:** ethylene signaling, ETR1, CTR1, EIN2, *Arabidopsis*

## Abstract

The gaseous plant hormone ethylene is perceived by a family of ethylene receptors and mediates an array of ethylene responses. In the absence of ethylene, receptor signaling is conveyed via the C-terminal histidine kinase domain to the N-terminus of the CONSTITUTIVE TRIPLE RESPONSE1 (CTR1) protein kinase, which represses ethylene signaling mediated by ETHYLENE INSENSITIVE2 (EIN2) followed by EIN3. In the presence of ethylene, the receptors are inactivated when ethylene binds to their N-terminal domain, and consequently CTR1 is inactive, allowing EIN2 and EIN3 to activate ethylene signaling. Recent findings have shown that the ethylene receptor N-terminal portion can conditionally mediate the receptor signal output in mutants lacking *CTR1*, thus providing evidence of an alternative pathway from the ethylene receptors not involving CTR1. Here we highlight the evidence for receptor signaling to an alternative pathway and suggest that receptor signaling is coordinated via the N- and C-termini, as we address the biological significance of the negative regulation of ethylene signaling by the two pathways.

## INTRODUCTION

**FIGURE 1:**
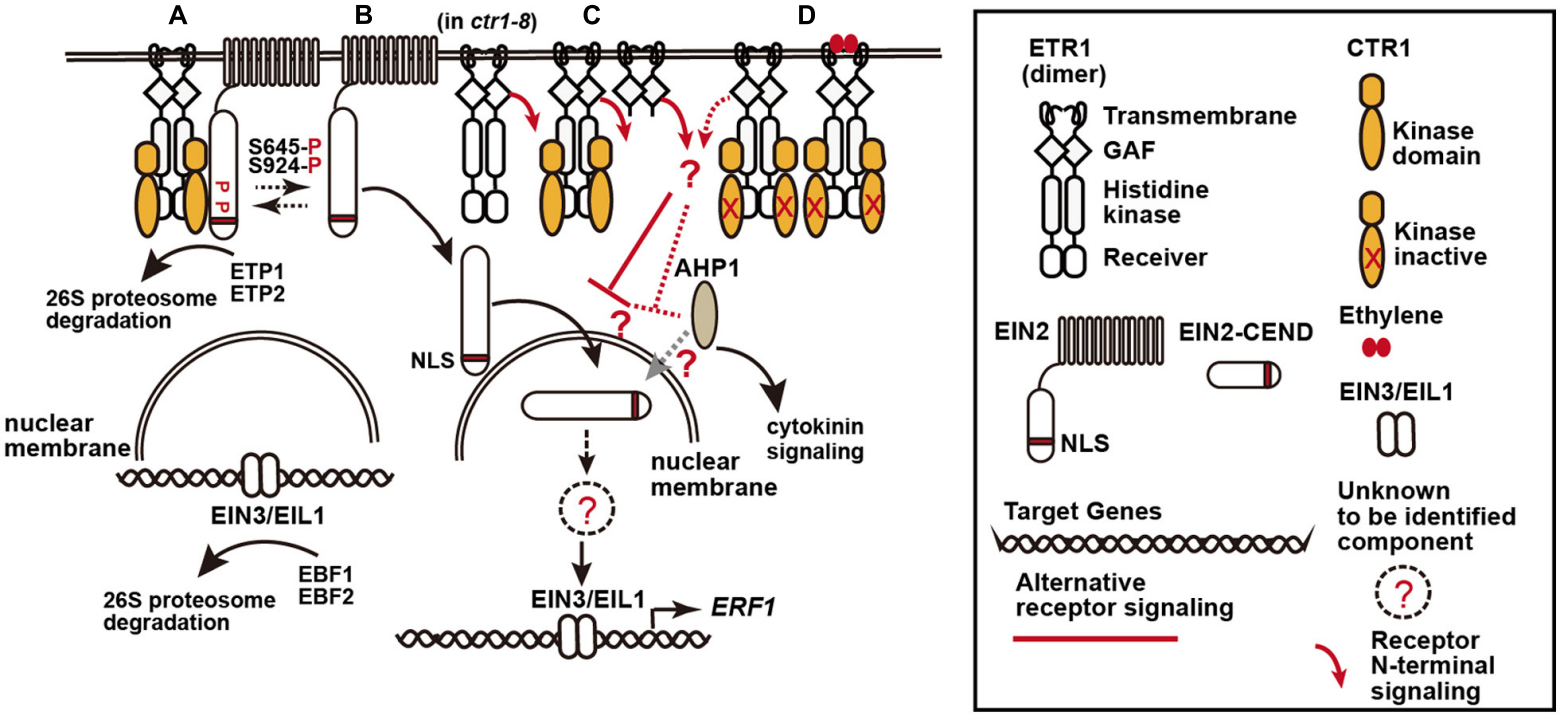
**A model of the ethylene receptor signaling via the C- and N-termini of ethylene receptor. (A)** Ethylene receptor C-terminal signaling, here represented by ETHYLENE RESPONSE1 (ETR1), is mediated via the histidine kinase domain to CTR1 to phosphorylate EIN2 residues S645 and S924, and EIN2 is retained at the endoplasmic reticulum for degradation mediated by the F-box proteins EIN2 TARGETING PROTEIN1 (ETP1) and ETP2. Ethylene signaling mediated by EIN2 to the nuclear EIN3/EIL1 is inhibited, and EIN3 and EIL1 undergo degradation mediated by the F-box proteins EIN3 BINDING F-BOX PROTEIN1 (EBF1) and EBF2. **(B)** “Default” ethylene signaling by EIN2. Without the receptors (in the receptor quintuple mutant), EIN2-mediated ethylene signaling fully occurs and the mutants produce an extremely strong constitutive ethylene response phenotype. With the receptors, a portion of EIN2 could be underphosphorylated, presumably because of interconversion between the phosphorylated and underphosphorylated state or incomplete phosphorylation by CTR1, and cleaved to potentially activate ethylene signaling. **(C,D)** Ethylene signaling suppression by the receptor N-terminus: **(C)** With the receptors, the “default” ethylene signaling mediated by EIN2 can be repressed in part by the receptor N-terminal signaling (red line), regardless of CTR1 docking. **(D)** Ethylene binding or docking of a kinase-inactive ctr1 at the receptors inhibits the receptor C-terminal signaling, and the cleaved EIN2 C-terminus is translocated to the nucleus to induce EIN3/EIL1-directed expression of ethylene response genes. The N-terminal signaling is also inhibited (red dotted line), and the inhibition of EIN2-mediated signaling is largely alleviated. Components involved in the receptor N-terminal signaling remain to be identified. AHP1 and EIN2 are proposed to be involved in the CTR1-independent pathway because of their interaction with ETR1, and AHP1 also mediates cytokinin signaling.

Signal transduction of the gaseous plant hormone ethylene has been studied for more than 2 decades, mainly with the dicotyledonous model plant *Arabidopsis*, and a linear signal transduction pathway has been proposed (**Figure 1A**; Ju and Chang, 2012; Ju et al., 2012; Shakeel et al., 2013). In the absence of ethylene, the ethylene receptors at the endoplasmic reticulum (ER) are active, and the docking of CONSTITUTIVE TRIPLE RESPONSE1 (CTR1) at the receptor histidine kinase domain facilitates CTR1 activation by unknown mechanisms. Serine/threonine kinase activity of CTR1 (Huang et al., 2003) results in phosphorylation of the C-terminal domain of ETHYLENE INSENSITIVE2 (EIN2; Ju et al., 2012). This phosphorylation prevents the EIN2 C-terminal domain from moving into the nucleus, and thusly prevents ethylene signaling. With ethylene binding to the receptors or in the absence of the receptors, CTR1 cannot be activated to phosphorylate EIN2. Underphosphorylated EIN2 undergoes proteolytic cleavage by an unknown mechanism to release a nuclear localization signal (NLS)-containing C-terminus, which enters the nucleus to mediate signaling to the EIN3 and EIN3-LIKE1 (EIL1) transcription factors. Such factors directly activate an array of primary ethylene response genes, including the *ETHYLENE RESPONSE FACTOR1* (*ERF1*) transcription factor gene (Chao et al., 1997; Solano et al., 1998; Qiao et al., 2012; Chang et al., 2013).

This model illustrates a framework of ethylene signaling; however, the dynamic fine-tuning of ethylene signaling remains to be fully addressed. One question we have been focusing on is a pathway that does not involve CTR1, as proposed 16 years ago (Hua and Meyerowitz, 1998). The finding that expression of the ethylene receptor ETHYLENE RESPONSE1 (ETR1) N-terminus represses ethylene signaling without involving CTR1 reveals an alternative pathway that greatly represses ethylene signaling (Gallie, 2012; Qiu et al., 2012). In this perspective article, we highlight studies that have revealed this alternative pathway for receptor signaling and discuss the dynamic coordination of the two pathways in negatively regulating ethylene signaling. We also discuss other alternative pathways that were have been previously suggested.

## EVIDENCE FOR ETHYLENE RECEPTOR SIGNALING NOT INVOLVING CTR1

Results from various studies imply that ethylene receptor signaling could be in part independent of CTR1. The *ctr1-1* mutation, encoding a D694E substitution, reduces CTR1 kinase activity to approximately <0.1% that of the wild-type activity and *ctr1-3*, encoding an R435STOP early termination, has a stronger phenotype than *ctr1-1* and the putative ctr1-3 protein does not have the kinase domain (Huang et al., 2003). The weak *in vitro* kinase activity of ctr1-1 that was detected could be due to the residual activity with the D694E substitution (Huang et al., 2003), non-specific phosphorylation, or a possible trace amount of kinase contamination during protein purification. Both *ctr1-1* and *ctr1-3* are responsive to ethylene treatment, indicating that ethylene receptor signaling can be mediated bypassing CTR1 (Larsen and Chang, 2001). A reciprocal evidence is the stronger constitutive ethylene response phenotype of the *Arabidopsis* quadruple and quintuple mutants (respectively, lacking 4 and 5 of the five homologous ethylene receptors) compared to the *ctr1-1* mutant (Hua and Meyerowitz, 1998; Liu et al., 2010).

It was considered that the loss of multiple ethylene receptors could impact seedling growth, and the severe mutant phenotype might not be solely due to strong constitutive ethylene responses (Hua and Meyerowitz, 1998). Nevertheless, the possibility of an alternative pathway was supported by experimental evidence showing that expression of *ETR1p:etr1^1-349^*, which encodes the ETR1 N-terminus (residues 1–349) lacking the CTR1 docking site, largely rescued the *ctr1-1* and *ctr1-2* mutant phenotypes and reduced the *ERF1* transcript level. The *ctr1-2* allele has a 17-bp deletion and probably encodes a truncated protein with 462 residues (Huang et al., 2003; Qiu et al., 2012), which was not immunologically detectable at the molecular-weight position of wild-type CTR1 (Gao et al., 2003). Ethylene insensitivity conferred by the dominant *etr1-1* mutation was prevented by *ctr1-1*, whereas expression of *ETR1p:etr1-1^1-349^* in *ctr1-1* conferred ethylene insensitivity. Moreover, expression of an *ETR1p:etr1^1-349^* transgene in the *etr1-1 ctr1-1* double mutant largely restored ethylene insensitivity conferred by the *etr1-1* allele, revealing the role of the ETR1 N-terminus in receptor cooperation and signal output without involving CTR1. Consistently, the *ctr1-1* allele prevents ethylene insensitivity by other ethylene-insensitive receptor genes, and ethylene insensitivity was restored to various degrees by the wild-type ETR1 N-terminus (Gallie, 2012; Qiu et al., 2012).

The CTR1 N-terminus is a regulatory domain that physically interacts with the ETR1 C-terminal histidine kinase domain (Clark et al., 1998). Excess CTR1 N-terminus (residues 7–560) generated by expression of a *35S:CTR1^7-560^* transgene, lacking the kinase domain, most likely occupies the ethylene receptors and prevents the normal receptor signal output, and the overexpressor (*CTR1-Nox*) consequently shows the typical constitutive ethylene response phenotype (Huang et al., 2003; Qiu et al., 2012). These studies define a role of the CTR1 N-terminus in receptor docking to mediate the receptor signaling to the CTR1 C-terminal kinase domain. However, there is more to this story. The constitutive ethylene response of *CTR1-Nox* was largely rescued by expression of the ETR1 N-terminus. etr1^1-349^ lacks the CTR1 docking site; without the docking of CTR1 N-terminus, the receptor N-terminal signaling may be mediated by an alternative pathway (Qiu et al., 2012).

Another line of evidence for the alternative pathway may come from studies of *ctr1-8*. In contrast to CTR1, which associates with ethylene receptors at the ER membrane, the ctr1-8 protein (with a G354E substitution) does not associate with ethylene receptors and is detected in the cellular soluble fraction (Gao et al., 2003; Huang et al., 2003). With these features, theoretically ctr1-8 cannot be activated by the receptors to phosphorylate EIN2, thus resulting in constitutive ethylene responses. However, the *ctr1-8* mutant has a relatively mild constitutive ethylene response phenotype as compared with *ctr1-1* and *ctr1-2* (Xie et al., 2012). Given that ctr1-8 has a wild-type kinase domain, the weak phenotype could be due to some ctr1-8 protein (below the limit of detection) still associating with the receptors to repress ethylene signaling. This scenario, however, is not supported by the result that overexpressing the N-terminus of wild-type CTR1 but not ctr1-8 increases ethylene sensitivity (Huang et al., 2003); whether the mutant ctr1-8 protein can still mediate receptor signaling is yet to be determined. Thus, the weak *ctr1-8* mutant phenotype could indicate suppression of ethylene signaling by the receptors without involving CTR1.

There are two models for an alternative ethylene signaling pathway, based on evidence from biochemical studies and protein–protein interactions. (1) Ethylene binding to the receptors results in the dynamic dissociation of CTR1 from, and association of EIN2 with, the receptors; in this case, the ethylene receptors might directly mediate ethylene signaling to EIN2 (Bisson and Groth, 2010, 2011). (2) Ethylene binding also facilitates the dissociation of the ETR1-interacting phosphotransfer protein *ARABIDOPSIS* HISTIDINE-CONTAINING PHOSPHOTRANSMITTER1 (AHP1) that is phosphorylated by ETR1 prior to ethylene binding (Scharein et al., 2008; Scharein and Groth, 2011). A conjectured two-component signaling model was proposed for ethylene signaling mediated by the receptors via AHPs to type B response regulator proteins known as *ARABIDOPSIS* RESPONSE REGULATORs (ARRs; Shakeel et al., 2013). *S*-nitrosylation of AHP1 Cys115 inhibits protein phosphorylation and subsequent phosphotransfer to ARR1, thereby suppressing cytokinin signaling (Feng et al., 2013). A complex formed by the two-component histidine kinase *ARABIDOPSIS* HISTIDINE KINASE5 (AHK5) and AHP1 is involved in a variety of biological processes (Bauer et al., 2013). Thus, AHP1 might differentially mediate signaling from various upstream histidine kinase proteins.

## ALTERNATIVE PATHWAY SUGGESTED BY *ctr1-1 EIN2/ein2*

**FIGURE 2:**
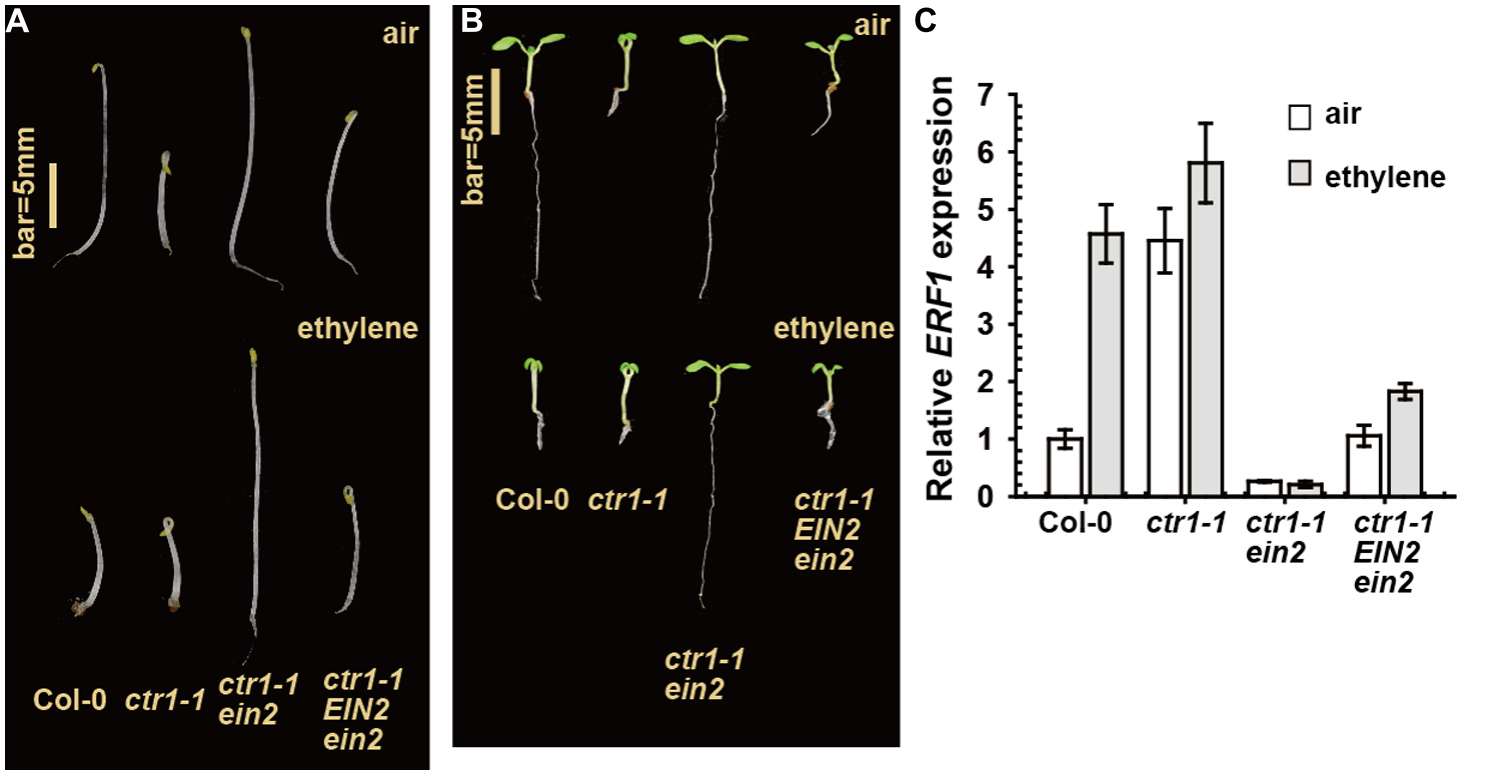
**The ethylene response phenotype of *ctr1-1 EIN2/ein2*.** The ethylene response phenotype for etiolated **(A)** and light-grown **(B)** seedlings and *ERF1* levels in rosettes **(C)**. Data are mean ± SE.

In a genetic screen for suppressors of *ctr1-1*, we isolated *ctr1-1 EIN2/ein2*, which showed a weaker constitutive ethylene response phenotype than *ctr1-1* (**Figure 2**). That is, with ethylene treatment, seedling hypocotyl elongation of this mutant (with *ein2* heterozygous) was inhibited to a lesser extent than that of ethylene-treated wild-type (Col-0) seedlings, with the *ctr1-1 ein2* seedling hypocotyl elongation unresponsive to ethylene (**Figure 2A**). Light-grown *ctr1-1 EIN2/ein2* seedlings produced a weaker growth-inhibition phenotype than *ctr1-1* with or without ethylene treatment, while *ctr1-1 ein2* seedling growth was unresponsive to ethylene (**Figure 2B**). The transcript level of the ethylene-dependent *ERF1* gene in the *ctr1-1 EIN2/ein2* mutant was slightly higher than in the *ctr1-1 ein2* double homozygote but much lower than in the *ctr1-1* mutant alone, and ethylene induction of *ERF1* expression was higher in *ctr1-1 EIN2/ein2* than *ctr1-1 ein2* but much lower than in the wild type (**Figure 2C**).

Sequence analysis revealed that a G1440A transition mutation caused a W308stop early termination of EIN2 in the newly isolated allele. The allele could conceivably produce a truncated EIN2 protein lacking the C-terminus for ethylene signaling and is likely a loss-of-function mutation. With a single-copy of wild-type *EIN2*, the EIN2 protein level in *ctr1-1 EIN2/ein2* may not be sufficient to fully induce the ethylene response to the same degree as *ctr1-1*. The mutation could be recessive or partially dominant because the heterozygote but not homozygote was responsive to ethylene. An important finding is that the *ctr1-1 EIN2/ein2* mutant appears to be slightly responsive to ethylene (**Figure 2**). This slight responsiveness to ethylene could reflect a pathway that does not involve CTR1. Without an alternative pathway to activate the wild-type copy of EIN2, the ethylene response would not be expected.

We therefore propose that ethylene binding could prevent the receptor N-terminal signaling so that ethylene signaling is relieved from suppression. Alternatively, ethylene signaling could be mediated by the receptors via AHPs to ARRs or directly to EIN2 (Bisson and Groth, 2011; Scharein and Groth, 2011; Shakeel et al., 2013).

## SIGNIFICANCE OF THE ALTERNATIVE PATHWAY IN ETHYLENE SIGNALING

Gene function can be partially inferred from the phenotype of loss-of-function mutants, and the effects conferred by different lesions of a gene may differ. The role of the alternative pathway in ethylene signaling could be minor given that *ctr1-1* and *ctr1-3* are slightly responsive to ethylene (Larsen and Chang, 2001) or the mutant ctr1-1 protein retains a trace of kinase activity (Huang et al., 2003). In contrast, the constitutive ethylene response phenotype is stronger in the ethylene-receptor quintuple mutant than in the *ctr1-1* mutant (Liu et al., 2010). The findings that *ctr1-8*, *ETR1p:etr1^1-349^ ctr1-2*, and *ctr1-1 EIN2/ein2* have a much weaker constitutive ethylene response phenotype than *ctr1-1*/*ctr1-2* suggest that the role of the alternative receptor-signaling pathway seems to be more pronounced than previously thought.

We explain these two conflicting interpretations for significance of the alternative pathway as follows. The inhibition of receptor signaling by the full-length receptors but not the truncated ETR1 N-terminus with expression of excess CTR1 N-terminus (Huang et al., 2003; Qiu et al., 2012) implies that the docking of a kinase-defective CTR1 protein at the receptors could inhibit the receptor N-terminal signaling to the alternative pathway (Xie et al., 2012). Conceivably, ctr1-1 and ctr1-2 could dock at the receptor C-terminus to inhibit the N-terminal signaling. In *ctr1-8*, the receptor N-terminal signaling to the alternative pathway is not inhibited, and the mutant produces a weak phenotype while being fully responsive to ethylene treatment that inhibits the N-terminal signaling. ETR1 and CTR1 may dissociate, with the Kd for ETR1- CTR1 interaction to be determined; the N-terminal signaling may not be fully inhibited by kinase-defective CTR1 proteins so that the constitutive ethylene response phenotype is weaker in *ctr1-1* and *ctr1-2* than the quintuple mutant, with *ctr1-1 EIN2/ein2* responsive to ethylene.

Whether the truncated ctr1-2 protein could dock at the receptors and ctr1-8 could mediate receptor signaling are to be demonstrated. More lines of experimental evidence are required to reveal the significance of the alternative ethylene signaling via the receptors to EIN2 or AHP1.

## DYNAMIC ETHYLENE RECEPTOR SIGNALING VIA THE C- AND N-TERMINI

A proposed model for the dynamic receptor ethylene signaling via the two termini is described in **Figure 1**. The model explains the following scenarios. (1) In the receptor quintuple mutants, the default “ethylene signaling” mediated by EIN2 is not affected because of the absence of the receptor C- and N-terminal signaling, and the mutant shows an extremely strong constitutive ethylene response phenotype (**Figure 1B**). (2) ctr1-8 does not dock at the receptors, and the N-terminal signaling is not inhibited; although the EIN2 C-terminus is theoretically released, ethylene signaling is nevertheless inhibited by the receptor N-terminal signaling, so that *ctr1-8* shows a relatively weak constitutive ethylene response phenotype (**Figure 1C**). (3) In mutants defective in CTR1 kinase activity (i.e., *ctr1-1* and *ctr1-2*), the mutant CTR1 proteins dock at the receptors to inhibit but not fully prevent receptor N-terminal signaling, whereas the EIN2 C-terminus is released for ethylene signaling (**Figure 1D**). Thus, the *ctr1* mutants produce a typical ethylene response phenotype that is weaker than the receptor quintuple mutant phenotype. (4) In *ctr1-1 EIN2/ein2*, receptor N-terminal signaling is inhibited; however, it is still sufficient to repress in part the ethylene signaling that is mediated by the EIN2 C-terminus, which exists at a reduced amount. Thus, the heterozygote shows a weaker phenotype than *ctr1-1*. Ethylene treatment inhibits the receptor N-terminal signaling, and the mutant is ethylene responsive.

## CONCLUSION AND REMARKS

Little is known about the components involved in the possible alternative pathway of ethylene perceived by ethylene receptors and mediation of receptor N-terminal signaling; mutants isolated from an ongoing suppressor screen in our laboratory for *ETR1p:etr1-1^1-349^ ctr1-1* could potentially isolate the involved components. The isolation of several *ENHANCED ETHYLENE RESPONSE* (*EER*) genes suggests a resetting mechanism damping ethylene signaling by components involved in various biological processes (Larsen and Chang, 2001; Robles et al., 2007; Christians et al., 2008; Deslauriers and Larsen, 2010; Lu et al., 2010). REVERSION-TO-ETHYLENE SENSITIVITY1 (RTE1) is an ER- and Golgi-associated protein facilitating ETR1 receptor N-terminal signaling (Resnick et al., 2006; Zhou et al., 2007; Gallie, 2012; Qiu et al., 2012), with its functions involving cytochrome b (Chang et al., 2014). The RTE1–cytrochrome b interaction may have a role in the alternative pathway. Unlike the ETR1 N-terminal signaling, the conjectured two-component signaling via AHPs and ARRs (Scharein and Groth, 2011; Shakeel et al., 2013) is presumably mediated via the receptor C-terminal histidine kinase domain. With a long half-life for ethylene binding to the receptors, a desensitizing mechanism is expectedly required for sustained AHP1 phosphorylation and dissociation from ETR1 and for EIN2 recruitment for ethylene signaling. The possibility for regulating ethylene signaling by multiple pathways or components is therefore likely.

Ethylene receptor signaling via the C- and N-termini could act independently, with distinct signaling components and targets; alternatively, the signaling could be mediated to different components, converged at the same signaling component, and sharing a common downstream pathway. EIN2 might mediate ethylene signaling via the receptor N-terminus because ETR1 and EIN2 physically interact and a single copy of the *ein2^G1440A^* allele partially suppressed *ctr1-1*; evidence for an interaction of ETR1 N-terminus with EIN2 is required to strengthen this scenario. EIN3 and EIL1 are the prime transcription factors responsible for inducing an array of ethylene response genes (Chao et al., 1997; An et al., 2010; Chang et al., 2013). The two pathways could converge and share a common signaling pathway that involves EIN3 and EIL1.

The signaling components may be dynamically present in a pathway for an immediate response. EIN2 could be present in a steady-state interconversion between the phosphorylated and underphosphorylated state in the absence of ethylene treatment; alternatively, a small portion, but not most, of EIN2 could escape the phosphorylation by CTR1 to ensure immediate ethylene signaling. Conceivably, a level of constitutive ethylene signaling could occur, if not repressed, to trigger stronger ethylene signaling. Alternative receptor signaling could have a role in minimizing degrees of the basal-level ethylene signaling. The two-level control may facilitate a dynamic fine-tuning of ethylene signaling in response to a wide range of ethylene concentrations.

## AUTHOR CONTRIBUTIONS

Jing Yu isolated the heterozygous *EIN2/ein2 ctr1-1* mutant, Jingyi Zhang characterized the ethylene response, and Chi-Kuang Wen wrote the paper.

## Conflict of Interest Statement

The authors declare that the research was conducted in the absence of any commercial or financial relationships that could be construed as a potential conflict of interest.

## References

[B1] An F. Zhao Q. Ji Y. Li W. Jiang Z. Yu X. (2010). Ethylene-induced stabilization of ETHYLENE INSENSITIVE3 and EIN3-LIKE1 Is mediated by proteasomal degradation of EIN3 binding F-Box 1 and 2 that requires EIN2 in *Arabidopsis*. *Plant Cell* 22 2384–2401 10.1105/tpc.110.076588 20647342 PMC2929093

[B2] Bauer J. Reiss K. Veerabagu M. Heunemann M. Harter K. Stehle T. (2013). Structure–function analysis of *Arabidopsis thaliana* histidine kinase AHK5 bound to its cognate phosphotransfer protein AHP1. *Mol. Plant* 6 959–970 10.1093/mp/sss126 23132142

[B3] Bisson M. M. A. Groth G. (2010). New insight in ethylene signaling: autokinase activity of ETR1 modulates the interaction of receptors and EIN2. *Mol. Plant* 3 882–889 10.1093/mp/ssq036 20591837

[B4] Bisson M. M. A. Groth G. (2011). New paradigm in ethylene signaling: EIN2, the central regulator of the signaling pathway, interacts directly with the upstream receptors. *Plant Signal. Behav.* 6 164–166 10.4161/psb.6.1.14034 21242723 PMC3122035

[B5] Chang J. Clay J. M. Chang C. (2014). Association of cytochrome b5 with ETR1 ethylene receptor signaling through RTE1 in *Arabidopsis*. *Plant J.* 77 558–567 10.1111/tpj.12401 24635651 PMC4040253

[B6] Chang K. N. Zhong S. Weirauch M. T. Hon G. Pelizzola M. Li H. (2013). Temporal transcriptional response to ethylene gas drives growth hormone cross-regulation in *Arabidopsis*. *Elife* 2 e00675. 10.7554/eLife.00675 PMC3679525 23795294

[B7] Chao Q. Rothenberg M. Solano R. Roman G. Terzaghi W. Ecker J. (1997). Activation of the ethylene gas response pathway in *Arabidopsis* by the nuclear protein ETHYLENE-INSENSITIVE3 and related proteins. *Cell* 89 1133–1144 10.1016/S0092-8674(00)80300-1 9215635

[B8] Christians M. J. Robles L. M. Zeller S. M. Larsen P. B. (2008). The eer5 mutation, which affects a novel proteasome-related subunit, indicates a prominent role for the COP9 signalosome in resetting the ethylene-signaling pathway in *Arabidopsis*. *Plant J.* 55 467–477 10.1111/j.1365-313X.2008.03521.x 18429939

[B9] Clark K. L. Larsen P. B. Wang X. Chang C. (1998). Association of the *Arabidopsis* CTR1 Raf-like kinase with the ETR1 and ERS ethylene receptors. *Proc. Natl. Acad. Sci. U.S.A.* 95 5401–5406 10.1073/pnas.95.9.5401 9560288 PMC20273

[B10] Deslauriers S. D. Larsen P. B. (2010). FERONIA is a key modulator of brassinosteroid and ethylene responsiveness in *Arabidopsis* hypocotyls. *Mol. Plant* 3 626–640 10.1093/mp/ssq015 20400488

[B11] Feng J. Wang C. Chen Q. Chen H. Ren B. Li X. (2013). S-nitrosylation of phosphotransfer proteins represses cytokinin signaling. *Nat. Commun.* 4 1529 10.1038/ncomms2541 23443557

[B12] Gallie D. (2012). F1000 prime recommendation of [Qiu L et al, *Plant Physiol.* 2012 159 (3):1263-76]. Faculty of 1000 10.3410/f.717953741.793459406.f1000.com/prime/717953741#eval793459406

[B13] Gao Z. Chen Y. F. Randlett M. D. Zhao X. C. Findell J. L. Kieber J. J. (2003). Localization of the raf-like kinase CTR1 to the endoplasmic reticulum of *Arabidopsis* through participation in ethylene receptor signaling complexes. *J. Biol. Chem.* 278 34725–34732 10.1074/jbc.M305548200 12821658

[B14] Hua J. Meyerowitz E. M. (1998). Ethylene responses are negatively regulated by a receptor gene family in *Arabidopsis thaliana*. *Cell* 94 261–271 10.1016/S0092-8674(00)81425-7 9695954

[B15] Huang Y. Li H. Hutchison C. E. Laskey J. Kieber J. J. (2003). Biochemical and functional analysis of CTR1, a protein kinase that negatively regulates ethylene signaling in *Arabidopsis*. *Plant J.* 33 221–233 10.1046/j.1365-313X.2003.01620.x 12535337

[B16] Ju C. Chang C. (2012). Advances in ethylene signaling: protein complexes at the endoplasmic reticulum membrane. *AoB Plants* 2012 pls031. 10.1093/aobpla/pls031 PMC3485614 23119138

[B17] Ju C. Yoon G. M. Shemansky J. M. Lin D. Y. Ying Z. I. Chang J. (2012). CTR1 phosphorylates the central regulator EIN2 to control ethylene hormone signaling from the ER membrane to the nucleus in *Arabidopsis*. *Proc. Natl. Acad. Sci. U.S.A.* 109 19486–19491 10.1073/pnas.1214848109 23132950 PMC3511113

[B18] Larsen P. B. Chang C. (2001). The *Arabidopsis* eer1 mutant has enhanced ethylene responses in the hypocotyl and stem. *Plant Physiol.* 125 1061–1073 10.1104/pp.125.2.1061 11161061 PMC64905

[B19] Liu Q. Xu C. Wen C.-K. (2010). Genetic and transformation studies reveal negative regulation of ERS1 ethylene receptor signaling in *Arabidopsis*. *BMC Plant Biol.* 10:60 10.1186/1471-2229-10-60 PMC2923534 20374664

[B20] Lu Q. Tang X. Tian G. Wang F. Liu K. Nguyen V. (2010). *Arabidopsis* homolog of the yeast TREX-2 mRNA export complex: components and anchoring nucleoporin. *Plant J.* 61 259–270 10.1111/j.1365-313X.2009.04048.x 19843313

[B21] Qiao H. Shen Z. Huang S.-S. C. Schmitz R. J. Urich M. A. Briggs S. P. (2012). Processing and subcellular trafficking of ER-tethered EIN2 control response to ethylene Gas. *Science* 338 390–393 10.1126/science.1225974 22936567 PMC3523706

[B22] Qiu L. Xie F. Yu J. Wen C.-K. (2012). *Arabidopsis* RTE1 Is essential to ethylene receptor ETR1 amino-terminal signaling independent of CTR1. *Plant Physiol.* 159 1263–1276 10.1104/pp.112.193979 22566492 PMC3387708

[B23] Resnick J. S. Wen C.-K. Shockey J. A. Chang C. (2006). From the cover: REVERSION-TO-ETHYLENE SENSITIVITY1, a conserved gene that regulates ethylene receptor function in *Arabidopsis*. *Proc. Natl. Acad. Sci. U.S.A.* 103 7917–7922 10.1073/pnas.0602239103 16682642 PMC1458508

[B24] Robles L. M. Wampole J. S. Christians M. J. Larsen P. B. (2007). *Arabidopsis* enhanced ethylene response 4 encodes an EIN3-interacting TFIID transcription factor required for proper ethylene response, including ERF1 induction. *J. Exp. Bot.* 58 2627–2639 10.1093/jxb/erm080 17526916

[B25] Scharein B. Groth G. (2011). Phosphorylation alters the interaction of the *Arabidopsis* phosphotransfer protein AHP1 with Its sensor kinase ETR1. *PLoS ONE* 6:e24173 10.1371/journal.pone.0024173 PMC3166298 21912672

[B26] Scharein B. Voet-Van-Vormizeele J. Harter K. Groth G. (2008). Ethylene signaling: identification of a putative ETR1–AHP1 phosphorelay complex by fluorescence spectroscopy. *Anal. Biochem.* 377 72–76 10.1016/j.ab.2008.03.015 18384742

[B27] Shakeel S. N. Wang X. Binder B. M. Schaller G. E. (2013). Mechanisms of signal transduction by ethylene: overlapping and non-overlapping signaling roles in a receptor family. *AoB Plants* 5 plt010. 10.1093/aobpla/plt010 PMC3611092 23543258

[B28] Solano R. Stepanova A. Chao Q. Ecker J. (1998). Nuclear events in ethylene signaling: a transcriptional cascade mediated by ETHYLENE-INSENSITIVE3 and ETHYLENE-RESPONSE-FACTOR1. *Genes Dev.* 12 3703–3714 10.1101/gad.12.23.3703 9851977 PMC317251

[B29] Xie F. Qiu L. Wen C.-K. (2012). Possible modulation of *Arabidopsis* ETR1 N-terminal signaling by CTR1. *Plant Signal. Behav.* 7 1243–1245 10.4161/psb.21545 22902695 PMC3493404

[B30] Zhou X. Liu Q. Xie F. Wen C.-K. (2007). RTE1 Is a golgi-associated and ETR1-dependent negative regulator of ethylene responses. *Plant Physiol.* 145 75–86 10.1104/pp.107.104299 17644624 PMC1976582

